# Improving Wearable-Based Activity Recognition Using Image Representations †

**DOI:** 10.3390/s22051840

**Published:** 2022-02-25

**Authors:** Alejandro Sanchez Guinea, Mehran Sarabchian, Max Mühlhäuser

**Affiliations:** Department of Computer Science, Technical University of Darmstadt, 64289 Darmstadt, Germany; me.srbn@gmail.com (M.S.); max@informatik.tu-darmstadt.de (M.M.)

**Keywords:** human activity recognition, image representation, CNNs, IMU, inertial sensors, wearable sensors

## Abstract

Activity recognition based on inertial sensors is an essential task in mobile and ubiquitous computing. To date, the best performing approaches in this task are based on deep learning models. Although the performance of the approaches has been increasingly improving, a number of issues still remain. Specifically, in this paper we focus on the issue of the dependence of today’s state-of-the-art approaches to complex ad hoc deep learning convolutional neural networks (CNNs), recurrent neural networks (RNNs), or a combination of both, which require specialized knowledge and considerable effort for their construction and optimal tuning. To address this issue, in this paper we propose an approach that automatically transforms the inertial sensors time-series data into images that represent in pixel form patterns found over time, allowing even a simple CNN to outperform complex ad hoc deep learning models that combine RNNs and CNNs for activity recognition. We conducted an extensive evaluation considering seven benchmark datasets that are among the most relevant in activity recognition. Our results demonstrate that our approach is able to outperform the state of the art in all cases, based on image representations that are generated through a process that is easy to implement, modify, and extend further, without the need of developing complex deep learning models.

## 1. Introduction

Ubiquitous computing has been envisioned as technology that provides support to everyday life, while disappearing into the background [1]. Disappearing into the background implies the goal of minimizing the technology’s intrusiveness and its demands of users [2]. A key aspect for supporting the everyday life of users is to be able to provide personalized services, for which is necessary to recognize what the user is doing. This task is known as Human Activity Recognition (HAR). The best performing approaches in that task to date are based on deep learning models. While the performance of the state-of-the-art approaches has been increasingly improving, a number of issues still remain. Specifically, we focus on the issue of the complexity of tuning or developing further the increasingly involved deep learning models considered for activity recognition, which incorporate ad hoc architectures of convolutional neural networks(CNNs), recurrent neural networks (RNNs), or a combination of both.

To cope with this issue in this paper we propose an approach that automatically transforms the inertial sensors time-series data into images that represent in pixel form patterns found over time, allowing even a simple CNN to outperform complex ad hoc deep learning models that combine RNNs and CNNs for activity recognition. Our approach can be considered as an extension of our preliminary workshop paper [3], where we introduce the concept of the image representation and present an initial evaluation with promising results. We have also presented in the past a similar approach but for the task of user identification [4]. In contrast to those two previous preliminary works, the current paper provides a clearer and more comprehensive rationale for the proposed image representations and details an image representation design and an image generation process that are more robust and capable of handling a wider range of scenarios. Furthermore, our evaluation in this case is much more extensive, comprising a wider range of experiments and configurations that allow to assess both the performance and the robustness of our proposed approach.

## 2. Related Work

### 2.1. CNNs for Time Series

In spite of the success that Convolutional Neural Networks (CNNs) have had in image processing tasks [5], due in part to their known capability of dealing with data with an intrinsic grid-like topology [6], a number of successful works have applied CNNs to time series data. In particular concerning ubiquitous computing, many works have proposed to use CNNs to deal with sensors time-series data (e.g., [7,8,9,10,11,12,13,14]). The majority of these previously proposed methods have been aimed at the task of activity recognition. In one of such works, a CNN is proposed to automate the feature learning from the raw sensor data in a systematic manner [9]. Another relevant related work uses a CNN combined with a long short-term memory (LSTM) network to extract time dependencies on features obtained by the CNN.

Similar to [7], the approaches in [12,13] combine CNNs with LSTMs. The approach in [13] uses a CNN to obtain high-level features, related to users’ behavior and physical characteristics. This is similar to the way the approach in [12] uses CNN for automated feature engineering. In [14], the authors use a distinct type of convolutional network, specifically a temporal convolutional network (TCN), which through a hierarchy of convolutions is able to entail the temporal relations of the time series data at different time scales.

The approaches mentioned above have used CNNs on time-series data in a direct way. That is, they have considered the time series as a one-dimensional grid, which is fed into the CNN directly. Instead, our approach creates 2D-image representations that entail patterns over time found in the time series. The resulting images are thus the input to a CNN model, with the intent of taking advantage of the strengths that CNNs have demonstrated when dealing with image processing tasks [5].

### 2.2. Image Representations of Time Series

A number of works have proposed to encode time series data into image representations, to then feed the images to a CNN to perform the classification (e.g., [15,16,17,18]). Specifically, in [15,16] the authors propose to encode time series using two different image representations: Gramian Angular Field (GAF) and the Markov Transition Field (MTF). The approach in [18] uses a similar encoding but for multiple time series. Other approaches have proposed to use a Short-time Fourier Transform (STFT) or Wavelet transform spectrogram to represent the time-series into image form (e.g., [19,20,21,22]).

Some approaches targeting HAR have proposed to form a 2D-image from the input signals (e.g., [23,24,25]). In these cases, the image formation consists simply in depicting the signals segments directly on an image. Therefore, it is as if the signals were imprinted on the images.

Approaches that simply depict the input signals directly on images (e.g., [23,24]) differ from our proposed method in that we first compute patterns over time from the time series data and then build an image that represents those patterns in a way that is better suited to be processed by a CNN, according to its widely recognized strengths, i.e., locality and edge detection [26]. On the other hand, approaches that have proposed an actual image representation, such as [15,18], use encodings that require relatively heavy computation, which make them unsuitable for real-time motion or action recognition [27]. In this respect, our approach is designed from the ground up to be easy to implement and represent a relatively low overhead.

## 3. Approach

Our approach is based on generating image representations from inertial sensors data. The generated images are then used as the input instances for a convolutional neural network (CNN) for activity recognition. Specifically, our image generation process is designed as to produce images that (a) take advantage of 2 well known strengths of CNNs, namely *locality* and *edge detection* [26], and (b) depict as many relevant patterns over time as possible in a single image.

### 3.1. Image Generation Process

**Figure 1:**
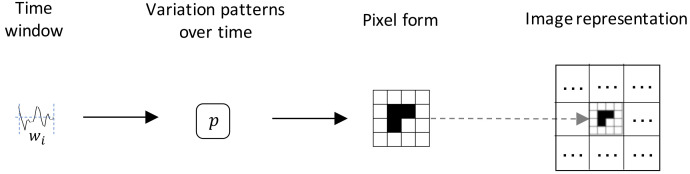
Image generation process.

To simplify the explanation, we first start with the case of one time window from an univariate time series and a single pattern. From the univariate time series, we take a time window $w_{i}$, from which we compute a pattern over time (see Section 3.2). We then transform the value of the pattern into pixel form (see Section 3.5), placing this on a specific region of the image (see Section 3.3). This process is illustrated in Figure 1. For a multivariate time series, each component of the time series is taken separately and represented in a different region of the image. The same is the case for neighboring time windows, i.e., each time window is represented in a specific region of the image. Following this process, it is possible to represent in a single-channel image as many patterns, time series components, and neighboring windows as needed by incorporating more regions of the image. Furthermore, color images (with 3 channels, i.e., RGB) allow to represent even more information on the regions that compose the extra channels (see Section 3.7). The time windows we consider are non-overlapping, as we found this to be the best configuration, since considering overlapping windows just slowed down the process without any evident improvement in performance.

### 3.2. Patterns over Time

We consider patterns that emerge over time in a single time window of an univariate time series. We decided for a set of patterns that are simple to compute and that can help effectively distinguish between different activities. Other methods in the literature have considered to either depict directly part of the signal on the image or depict the result of signal transformations such as Fourier Transform (e.g., [19]) or Wavelet Transform (e.g., [20,21,22]). In the first case, the design effort is placed on the neural network architecture, which is something we are seeking to avoid with our approach. Concerning the second case, the transformations used involve heavy computation [27], which stands in contrast to the simple computations needed in our case.

**Figure 2:**
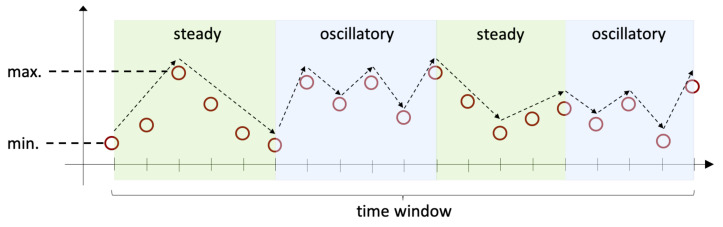
Variation patterns over time.

Specifically, the patterns we consider are:

*Oscillatory variation.* This pattern refers to the case in which the value of the data points in the time window go up and down from one time step to the next one. Specifically, if we consider 3 consecutive time steps $t_{i}$, $t_{j}$, and $t_{k}$, where $t_{i}<t_{j}<t_{k}$, we have either that at times $t_{i}$ and $t_{k}$ the values of the corresponding data points $d_{i}$ and $d_{k}$ are strictly higher than the value of the data point $d_{j}$ at time $t_{j}$, or that at time $t_{j}$ the value of the corresponding data point $d_{j}$ is strictly higher than both the values of the data points $p_{i}$ at $t_{i}$ and $p_{k}$ at $t_{k}$. This pattern is illustrated on the blue areas shown in Figure 2.
*Steady variation.* This pattern refers to the case where the value of the time window goes either up or down consistently. This is illustrated on the green areas in Figure 2.
*Range.* This pattern refers to the maximum, minimum, and the difference between maximum and minimum data point values within the time window.

As we can observe in Figure 2, within one time window it is possible to recognize more than one type of variation patterns. To compute the value of either variation pattern (vp), we consider all segments within the time window where the data points of the respective pattern are present and we use the formula in Equation (1), where $d_{i}$ corresponds to the value of the data point at time step $t_{i}$ and $x_{i-1}$ to the value of the data point at the previous time step, i.e., $t_{i-1}$. Note that since in order to recognize the type of variation pattern we need at least 3 data points, it is ensured that k≥3 in the equation.

(1) $$vp=\frac{1}{k-1}\sum_{i=1}^{k}\left|d_{i}-d_{i-1}\right|$$

### 3.3. Canvas & Regions [Locality]

**Figure 3:**
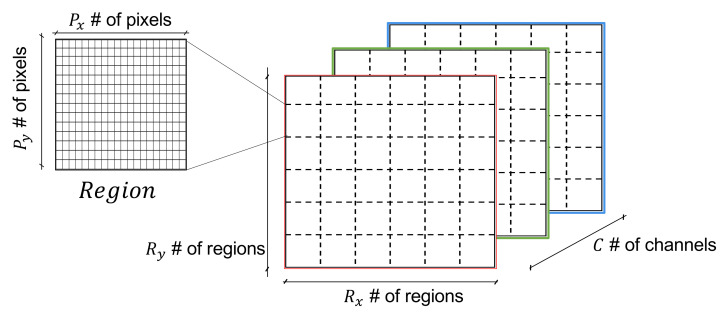
Canvas & Regions [locality].

In order to obtain images that purposely exploit the *locality* strength of CNNs, we seek to consistently represent specific pattern(s) in specific regions of the images. To this end, we consider an image as an empty ‘canvas’ or $R_{x}\times R_{y}$ number of regions and *C* number of channels. Black and white (B & W) images correspond to C=1, while color (RGB) images to C=3. Each region is composed of $P_{x}\times P_{y}$ number of pixels. A canvas divided into regions as explained is illustrated in Figure 3.

**Figure 4:**
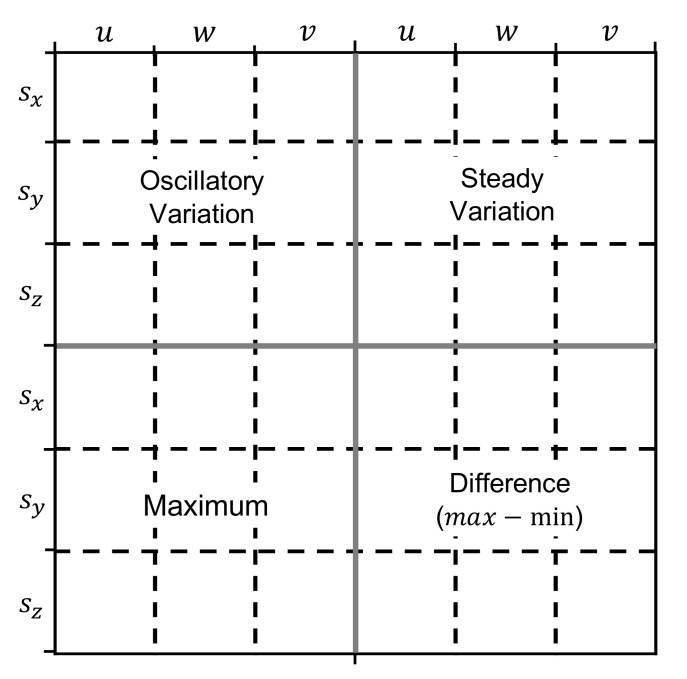
Canvas, quadrants, regions.

In this paper, we consider the following specific design for the images. The canvas is split into 36 regions (6×6) and these cover a total of 4 *quadrants*, which correspond to 9 regions (3×3). This canvas is depicted in Figure 4, where it can be seen that the oscillatory and steady variations are placed on the top-left and top-right quadrants, respectively. On the other hand, range patterns are placed at the bottom, namely the maximum is on the bottom left and the difference between maximum and minimum is on the bottom right.

We further enrich the image representations by including patterns found in neighboring time windows. In particular, as depicted in Figure 4, each region represents a pattern corresponding to one of the components of the inertial sensor ($s_{x}$, $s_{y}$, and $s_{z}$) for a specific window span (*u*, *w*, or *v*). In this case *w* corresponds to the current time window, *u* to the windows before the current window, and *v* to the windows after the current window. In this paper, we consider two windows for *u* and *v*, namely the regions covered by *u* correspond to patterns found in the two windows before the current one, and the regions covered by *v* refer to patterns found in the 2 windows after the current window.

### 3.4. Pixel Filling [Edge Detection]

**Figure 5:**
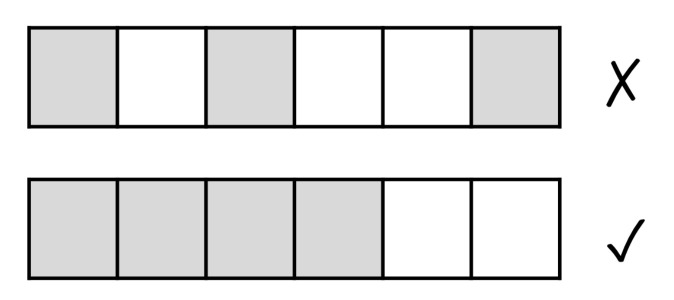
Continuous pixel marking (on the bottom) [edge detection].

To generate images that can exploit the edge detection strength of CNNs, we seek to produce clearly distinguishable edges that represent the patterns of interest in a consistent manner. To this end, we introduce the concepts of ‘marked’/‘unmarked’ pixels and of ‘continuous pixel marking’. A ‘marked’ pixel corresponds to the value 255 (i.e., white color), whereas an ‘unmarked’ pixel corresponds to the value 0 (i.e., black color). We have chosen the such contrast between ‘marked’ and ‘unmarked’ pixel to accentuate the edges produced. *Continuous pixel marking* refers to filling in the pixels in a region by marking them consecutively based on a specific ‘filling’ strategy (see Section 3.6). This is illustrated in Figure 5. As we can see in the figure, by using *continuous pixel marking* we ensure the generation of well-defined and continuous edges.

### 3.5. Mapping Inertial Sensors to Pixels

To obtain the number of ‘marked’ pixels corresponding to a given pattern value, we define a mapping function *M* as:

(2) M:patternvalue↦No.‘marked’pixels

To compute this we need to consider the minimum ($\min_{p}$) and maximum ($\max_{p}$) values of the given pattern for the current time window, and the minimum ($\min_{px}$) and maximum ($\max_{px}$) number of pixels that can potentially be ‘marked’ within a region. This allows to transform any given pattern value $p_{a}$ into the corresponding number of ‘marked’ pixels within a region, according to Equation (3).

(3) $$M\left(p_{a}\right)=\left\lfloor \min_{px}+\left(\frac{\max_{p}-\min_{p}}{\max_{px}-\min_{px}}\right)\left(p_{a}-\min_{p}\right)\right\rfloor$$

As it can be observed in Equation (3), the value of $M(p_{a})$ is ensured to be a whole number, since we are considering the floor function. In this way, the value is easily translatable into the number of ‘marked’ pixels. Any pixel that is no ‘marked’ is considered ‘unmarked’.

### 3.6. Filling Strategies

**Figure 6:**
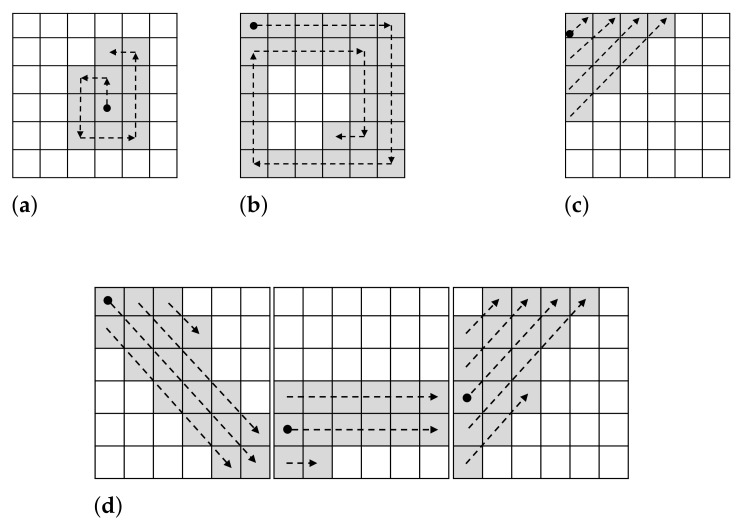
Filling strategies. (**a**) Counterclockwise (CCW); (**b**) Clockwise (CW); (**c**) Diagonal (Diag); (**d**) Strokes (Strk).

A number of different fillings strategies can be proposed. For this paper, we only focused on strategies aimed at producing images with well localized and distinguishable edges that can be easily reproduced. Next, we describe the filling strategies we consider, which are illustrated in Figure 6.

*Counterclockwise (CCW).* This strategy, which is illustrated in Figure 6a, starts at the center pixel of the region, or in the pixel right before the center in the case of a region with an even number of pixels. From that point, the ‘marked’ pixels are filled in continuously, first up one pixel, then left, then down, and so on in a counterclockwise manner. The path that it is followed is such that does not considers the same pixel twice and leaves no pixel ‘unmarked’ on the way.
*Clockwise (CW).* This strategy (illustrated in Figure 6b) starts at the pixel of the top-left corner of the region. The path then makes pixels ‘marked’ in a clockwise fashion, while avoids considering the same pixel twice or skipping any pixel.
*Diagonal (Diag).* This strategy that is depicted in Figure 6c has the pixel at the top-left corner of the region its starting point. The path from there corresponds to a 45° diagonal that goes upwards. The pixels are never considered twice and the pixels on the path are never left ‘unmarked’.
*Strokes (Strk).* This strategy (see Figure 6d) seeks to produce continuous ‘strokes’ that pass over 3 regions of the same row of the image representation. To do this, we consider for each of the regions on the row diagonal or horizontal lines, which are then followed on the next region by a different type of line. That is, the path considered in the first region on the left is of reverse diagonals. This is then followed in the next region by a path of horizontal lines. Finally, the last region considers a path of diagonal lines. The lines are drawn from left to right and are stacked one on top and one on the bottom of the latest line. By default we consider the top-left corner the beginning of the path. This is true for the first left-most region and for any region where no pixel has been marked in the previous region to the left that is directly adjacent to the region under consideration. If the case is different, then the starting point considered is the one next to the highest ‘marked’ pixel in the previous region to the left which is directly adjacent to the region under consideration. In Figure 6d an example of this strategy is shown. As it can be seen, the left-most region starts at the top-left corner (depicted by a black dot) and it goes into filling that reverse diagonal, then the one on the top of that, then the one on the bottom of the first one, and finishes half-way through the diagonal on top of the second diagonal. The next region to the right then starts at the pixel that is adjacent to the highest ‘marked’ pixel in the previous region, using horizontal lines from left to right, and filling next the line on top of the first one and then the line below. The third region is filled in a similar way, but following diagonal lines.

### 3.7. Coloring

In order to incorporate further information as part of the image representations we consider the three channels of a color (RGB) image, namely red, green, and blue. In this paper, we propose two ways of using the color channels. One option is to represent patterns found in time windows next to the current time window. The second option we propose is to depict patterns from multiple sensors, using each color channel to represent the patterns of a different sensor.

#### 3.7.1. Color Channels to Represent Nearby Time Windows

**Figure 7:**
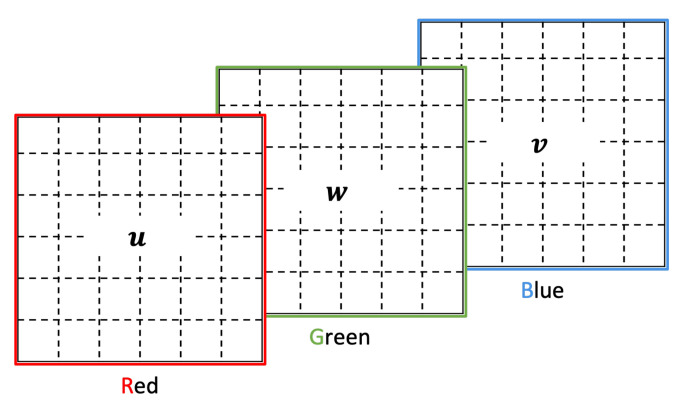
Simple color channel assignment to represent nearby time windows patterns.

For this option we propose two different alternatives. The first one, depicted in Figure 7, is a simple assignment of the current window *w*, the window(s) *u* before the current one, and the window(s) *v* following the current one to each of the color channels.

**Figure 8:**
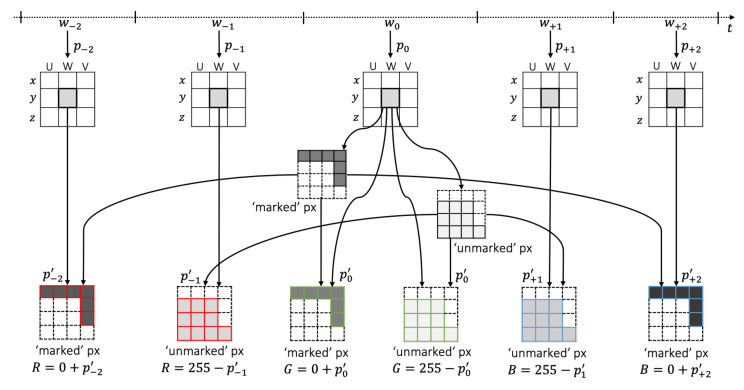
‘marked’ and ‘unmarked’ coloring based on nearby windows.

The second option we propose to represent neighboring time windows patterns is aimed at representing in a different manner the window $w_{-1}$, which is right before the current window than the window $w_{-2}$, which precedes $w_{-1}$, and similarly for the windows after the current one. Specifically, for this case all channels use the same canvas with the same ‘marked’ and ‘unmarked’ pixels, obtained as we explained previously. However, the specific pixel value of each of the ‘marked’ and ‘unmarked’ pixels is based on the previous windows (i.e., $w_{-1}$ and $w_{-2}$) for the red channel, on the current window $w_{0}$ for the green channel, and on the following windows (i.e., $w_{+1}$ and $w_{+2}$) for the blue channel. The purpose of this is to encode in the color of the image as much information as possible of what can be found in the neighbor time windows of the current one. The red channel’s ‘marked’ pixels are derived from considering the value of the pattern $p_{-2}$ corresponding to the same area for the image representing one window before the previous (i.e., $w_{-2}$) scaled within the range of color values [0,255]. This is $p_{-2}^{\prime }$. After that, the pixel value is obtained by adding $p_{-2}^{\prime }$ to the minimum possible pixel value (i.e., 0) as $R_{‘marked’}=0+p_{-2}^{\prime }$. The process for the red channel’s ‘unmarked’ pixels, on the other hand, considers the pattern corresponding to the immediate previous window (i.e., $p_{-1}$), which is scaled within the range of possible pixel values [0,255] to obtain $p_{-1}^{\prime }$. Then, the pixel value is obtained by subtracting $p_{-1}^{\prime }$ from the maximum possible pixel value (i.e., 255) as $R_{‘unmarked’}=255-p_{-1}^{\prime }$. The pixel value for the green channel is obtained considering the scaled pattern value of the current window $p_{0}^{\prime }$ as $G_{‘marked’}=0+p_{0}^{\prime }$ for ‘marked’ pixels and $G_{‘unmarked’}=255-p_{0}^{\prime }$ for ‘unmarked’ pixels. Finally, the pixel values for the blue channel’s ‘marked’ and ‘unmarked’ pixels are obtained as $B_{‘unmarked’}=255-p_{+1}^{\prime }$ and $B_{‘marked’}=0+p_{+2}^{\prime }$, respectively. This process is illustrated in Figure 8, focusing only on one specific region in the middle of a particular quadrant within an image representation. As it can be observed, all the channels in the color image at the bottom of the figure share the same ‘marked’ and ‘unmarked’ pixels, which follows the configuration found in the image representing $w_{0}$. The difference is only on the pixel value that each of the ‘marked’ and ‘unmarked’ pixels will have on each of the channels.

#### 3.7.2. Color Channels to Represent Multiple Sensors

**Figure 9:**
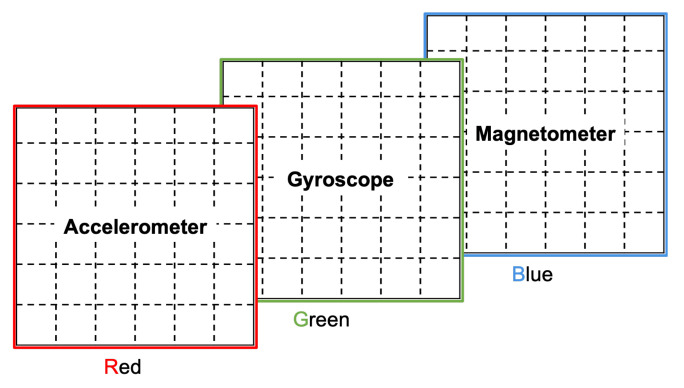
Simple color channel assignment to represent multiple sensors.

In this option, the patterns of each inertial sensor is represented on a different color channel, as illustrated in Figure 9. This can be a good option when dealing with inertial measurement units (IMUs), which typically contain an accelerometer, a gyroscope, and a magnetometer. However, it has the clear limitation imposed by the number of available color channels, which makes it unsuitable when considering more than 3 inertial sensors. For such a case, we propose below the augmentation of the canvas.

### 3.8. Augmented Canvas for Multiple Sensors

**Figure 10:**
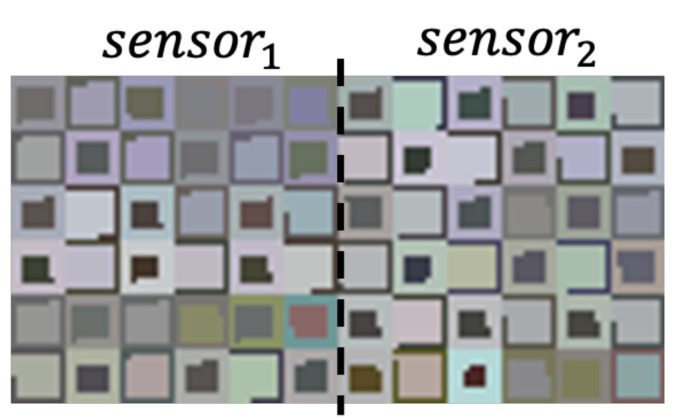
Augmented canvas for multiple sensors.

To incorporate patterns from more than one sensor, we consider images of larger size, which are obtained by placing side by side images generated from different sensors. This is illustrated in Figure 10, where we see a sample of an image generated with our approach for different the USC-HAD dataset.

## 4. Evaluation Methodology

To evaluate our approach, we consider 7 of the most widely recognized datasets in HAR [28], comparing for each dataset the performance of our method against the methods in the literature that we found have achieved highest performance. We provide the comparison with respect to at most 5 other approaches with best performance in the literature for each dataset. Specifically, the datasets we consider are WISDM, UCI-HAR, USC-HAD, PAMAP2, Opportunity, Daphnet, and Skoda. In each case, we configure our image representations to make use of the same inertial sensors considered by the state-of-the-art approaches and measure the performance using the same metrics they use. Concerning the machine learning evaluation, to ensure that the comparison is fair, we consider the same metric as the one used in the paper where the approach was proposed and evaluated. Furthermore, the data split we use on each case corresponds to the one considered by what we found to be the best performing approach for the corresponding dataset. As a baseline we consider a simple convolutional neural network (CNN) defined as follows. Two convolutional layers, each considering 128 filters of size 3 × 3. Each convolutional layer is followed by a max-pooling layer, with filter size of 2 × 2 and a stride of 2. These layers are followed by a dense layer of 256 units. Furthermore, we use softmax activation on the last layer, ReLU on the intermediate layers, cross-entropy loss function, Adam optimizer, and early stopping. For the image generation process, we consider in all cases CCW and CW filling strategies for each region in an iterative sequence (i.e., CCW, CW, CCW, …). The coloring strategy we used is the one illustrated in Figure 8. We decided to use this design as we found it to be the best one in terms of the results yielded. The implementation was made using the Keras library, https://keras.io/ (accessed on 27 December 2021).

### 4.1. Datasets

#### 4.1.1. WISDM Dataset

**Figure 11:**
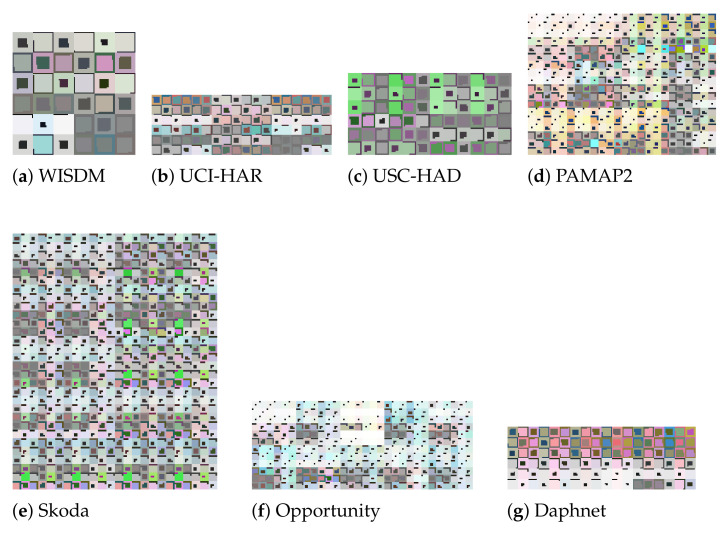
Samples of the image representations: (**a**) WISDM (120 × 120 px.), (**b**) UCI-HAR (180 × 60 px.), (**c**) USC-HAD (120 × 60 px.), (**d**) PAMAP2 (120 × 90 px.), (**e**) Skoda (120 × 150 px.), (**f**) Opportunity (150 × 60 px.), and (**g**) Daphnet (180 × 60 px.)

This dataset https://www.cis.fordham.edu/wisdm/dataset.php (accessed on 27 December 2021) was collected from a smartphone, only considered the accelerometer. In [29], was the first time that the dataset was introduced and detailed. A total of 36 users were considered for the collection, where each user followed 6 activities: going up and down the stairs, jogging and walking, as well as standing. Figure 11a depicts a sample of an image representation that our approach generated for this dataset. For the machine learning evaluation we consider a 10-fold cross-validation, as described in what we found to be the best performing approach in the literature for this dataset, namely Hossain et al. [30].

#### 4.1.2. UCI-HAR Dataset

This dataset https://archive.ics.uci.edu/ml/datasets/human+activity+recognition+using+smartphones (accessed on 27 December 2021) was also collected data using a smartphone. In this case the smartphone was waist-mounted and the data recorded corresponds to the accelerometer and the gyroscope in the smartphone. In [31], the authors introduced first this dataset, which collected data from 30 users. The image generation considered in this case took the time series corresponding to acceleration from the accelerometer, estimated body acceleration, and angular velocity from the gyroscope. As mentioned in Section 3.8, when considering multiple inertial sensors, we put together in a single image each of the images generated from every single sensor’s data. Figure 11b shows a sample of an image representation that was generated by our approach from this dataset. For the machine learning evaluation, we consider 10-fold cross validation, as it was considered by what we found to be the best performing approach in the literature for this dataset, i.e., Ignatov [24].

#### 4.1.3. USC-HAD Dataset

This dataset http://sipi.usc.edu/had/ (accessed on 27 December 2021) considers 12 simple activities, such as walking, running, jumping, sitting, standing, and sleeping. The number of participants for the data collection was 14. The inertial sensors used were accelerometer and gyroscope, which are the ones used for the image generation in our case. A sample of the image representations generated for this dataset can be seen in Figure 11c. The machine learning evaluation in this case is made considering a random 80% (training), 20% (testing) split, as described in what we found to be the best performing approach for this dataset, namely Murad and Pyun [32].

#### 4.1.4. PAMAP2 Dataset

This dataset http://www.pamap.org/demo.html (accessed on 27 December 2021) considers 3 IMUs places on the chest, arm and ankle of a total of 9 participants. The activities performed were 18. The generated images for this dataset place side by side 12 images, each of which was generated for each IMU. In Figure 11d, we can observe a sample of an image representation generated from this dataset. For the machine learning evaluation we consider a random 80% (training), 10% (validation), and 10% (testing) split, as described in what we found to be the best performing approach for this dataset, namely Jafari et al. [25].

#### 4.1.5. Opportunity Dataset

We consider the Opportunity ‘challenge’ dataset http://www.opportunity-project.eu/challengeDataset.html (accessed on 27 December 2021) first used in [33]. The data considers 4 participants performing 6 different iterations of broad morning routines and a set of specific activities such as opening and closing the fridge, opening and closing the dishwasher, turn on and off the lights, drinking, and cleaning the table. The dataset was recorded with each participant wearing 12 accelerometers in different parts of their body. The image representations for this dataset are obtained by placing side by side the images generated from 10 of the sensors. The reason to consider only 10 out of the 12 accelerometers is to have a similar configuration with respect to the state-of-the-art approaches against which we compare the performance of our approach. A sample of a generated image for this dataset is shown in Figure 11f. The machine learning evaluation in this case was performed using a 10-fold cross-validation, as described in what we found to be the best performing approach in the literature for this dataset, namely Hossain et al. [30].

#### 4.1.6. Daphnet Freezing of Gait Dataset

This dataset https://archive.ics.uci.edu/ml/datasets/Daphnet+Freezing+of+Gait (accessed on 27 December 2021) considers 10 subjects wearing 3 accelerometers. The goal is to detect sudden and transient inability to move, known as Freeze of Gait, while the participants walk [34]. The image representations in this case are generated by concatenating three image representations, one for each of the three accelerometers. A sample of an image representation for this dataset is depicted in Figure 11g. The split used for the machine learning evaluation in this case was 10-fold cross-validation, as described by what we found to be the best performing approach for this dataset, i.e., Hossain et al. [30].

#### 4.1.7. Skoda Mini Checkpoint Dataset

This dataset http://har-dataset.org/lib/exe/fetch.php?media=wiki:dataset:skodaminicp:skodaminicp_2015_08.zip (accessed on 27 December 2021) was collected from 8 subjects performing 10 times a car checking procedure. Each participant performed the tasks while wearing a special motion jacket with 7 IMUs. The dataset was first introduced and used in [35]. Our image representations in this case are generated based on data 16 inertial sensors, in order to have a fair comparison to the approaches against which we compare our approach’s performance. A sample of an image representation for this dataset is depicted in Figure 11e. For the machine learning evaluation we consider a random 80% (training), 10% (validation), and 10% (testing) split, as described in what we found to be the second-best performing approach for this dataset in the literature, namely Zeng et al. [36]. We consider the second-best because the best performing approach in this case, did not mention the split used.

### 4.2. Metrics

We consider standard metrics used to measure the performance of machine learning classification approaches, according to what the state-of-the-art methods in our benchmark have used. In a basic binary classification one may have the following basic options: (a) *true positives (TP)* when the model correctly predicts the positive class, (b) *false positives (FP)* when the model mistakenly predicts the positive class, (c) *false negatives (FN)* when the model wrongly predicts the negative class, and (d) *true negative (TN)* when the model correctly predicts the negative class. Based on this, we define the metrics Accuracy (Equation (4)), Precision (Equation (5)), Recall (Equation (6)) and $F_{1}$-score (Equation (7)) used in our evaluation in a per-class basis.

(4) $$Accuracy=\frac{TP+TN}{TP+TN+FP+FN}$$

(5) $$Precision=\frac{TP}{TP+FP}$$

(6) $$Recall=\frac{TP}{TP+FN}$$

(7) $$F_{1}\text{-}\mathrm{score}=2*\frac{Precision\times Recall}{Precision+Recall}$$

## 5. Results

### 5.1. Benchmarking

In this section, we conduct a comparison of the performance of our approach against the best performing approaches we were able to find in the literature. We first focus on other approaches that also proposed image representations for HAR. Then, we compare the performance of our approach against the results reported in the papers that have introduced the best performing approaches that we could find in the literature, for each of the datasets we consider.

#### 5.1.1. Comparison to Existent Image Representations

We were able to find three existing approaches in the literature that have proposed image representations for HAR (i.e., [23,24,25]). Table 1 shows a comparison between the performance of our approach and each of these three existing approaches. The comparison is made considering the same datasets used in the papers where each of these related works were introduced. Furthermore, we consider the same metric as the one considered in each respective paper, seeking to obtain a fair comparison. Likewise, the data split we use for machine learning evaluation on each case, corresponds to the one considered by what we found to be the best performing approach for the corresponding dataset. The results of these works are taken directly as presented by their authors.

As we can see in Table 1, in terms of performance, our approach is able to outperform these existing methods in all the cases considered. Furthermore, our approach is only concerned with the construction of image representations, which does not require any specialized deep learning expertise or involvement for implementation or further development, whereas the other methods propose both an image representation and a specific CNN architecture (e.g., Sensornet architecture [25]).

#### 5.1.2. Comparison to Best Performing Approaches for Each Dataset

In this section, we present the comparison between the performance of our approach and the best performing approaches in the literature, for each of the 7 datasets that we consider. The performance result from the state-of-the-art approaches is reported exactly as it appears on the paper where the approach was first introduced.

##### WISDM Dataset

The results of the evaluation of our approach using this dataset are presented in Table 2. As we can see, our approach shows a higher accuracy with respect to all state-of-the-art approaches. In all cases, the approaches under consideration use neural networks, particularly CNNs. In particular, in [24] time series data is treated as image data and passed through a CNN, in [37,38] the authors propose a new CNN architecture to deal with HAR, and in [19] the authors combine deep learning models with spectrograms and Hidden Markov Models (HMM) to perform the activity recognition. Among the approaches considered, it is worth noting that our approach is able to outperform the approach in [30], which it is an approach that uses user input through active learning to improve the accuracy.

##### UCI-HAR Dataset

The results of the evaluation of our approach using this dataset are presented in Table 3. Our approach clearly outperforms the state of the art when considering this dataset. The state-of-the-art approaches include in this case an end-to-end deep learning model [40], a method that stacks the raw signals from the inertial sensors into images which serve as the input instances to a CNN [23], a method that treats time series data as image data [24], and an approach that uses directly the time series as the input of the CNN [41].

##### USC-HAD Dataset

The comparison of our results against the results of the state of the art for this dataset are presented in Table 4. As we can see, the performance of our approach is higher that the one of the existing methods. Interestingly, for this dataset we get to compare our approach to two methods that consider Long short-term memory (LSTM) models (i.e., [32,42], which are recurrent neural networks that have been proposed particularly to deal with time series data. In any case, our approach manages to outperform all existing methods in the state of the art.

##### PAMAP2 Dataset

The comparison of our results against the results of the state of the art for this dataset are presented in Table 5, where we can see that our approach has a higher performance overall. The state of the art in this case includes an approach that consider LSTM models [36], two approaches that propose the use of a combination of a CNN and a recurrent model to account for both spatial and temporal patterns (i.e., [10,43]), and an approach that transforms multimodal time series into images that are then used as input to a CNN [25]. Although this approach also considers image representations of time series, our results show that our encoding achieves a better accuracy.

##### Opportunity Dataset

The comparison of our results against the results of the state of the art for this dataset are presented in Table 6. As it can be observed, our approach outperforms the state of the art, which includes approaches that consider LSTM models alone [10] and in combination to CNNs [7,44], as well as an approach that considers the active input from users to improve the performance [30].

##### Daphnet Freezing of Gait Dataset

The comparison of our results against the results of the state-of-the-art approaches for this dataset are presented in Table 7. Our approach is able to outperform the best performing exiting approaches for this dataset, including an approach that considers data from multiple sensors and the machine learning concept of attention to improve performance [45], an end-to-end deep learning approach [40], and an approach to deal with HAR based on active learning [30].

##### Skoda Mini Checkpoint Dataset

The comparison of our results against the results of the state-of-the-art approaches for this dataset are presented in Table 8. The approaches in the literature with highest performance for this dataset include an ensemble method that obtains its results best on the best results from a set of LSTM models [46], approaches that consider the machine learning concept of attention to improve performance [36,47], and an end-to-end approach that combines convolutional networks and LSTM models to account for the spatio-temporal patterns in the data [7]. In all cases, our approach is able to outperform their performance.

#### 5.1.3. Summary of Benchmarking

In Table 9, we summarize the overall results of our evaluation, where we present the performance of our approach against the results of the best performing approach in the literature for each of the 7 datasets we consider.

We can make *two main observations* about the results in Table 9 and in general of the comparison we have made with respect to all state-of-the-art approaches in the literature. The first one is that our approach is able to outperform each of the best performing approaches for all the datasets we consider. While the difference between our results and the existent approaches is not substantial (*avg.* = 0.0278), it provides evidence that overall our approach is sound and capable of handling a wide variety of datasets, maintaining a top performance. The second observation is that compared to our approach, all the state-of-the-art approaches are making use in general more sophisticated machine learning methods or consider more complex or elaborate neural network architectures. One approach considers active learning during the fine tuning phase in order to single out the most informative data instances [30]. Two other approaches proposed the use of neural network architectures based on U-Net [39], which contain large amounts of layers (23 conv. layers [38] and 28 conv. layers [48]). With the goal of accounting for temporal information, 5 approaches propose the use of LSTMs, either alone [10,32,42] or in combination with CNNs [7,43,44]. All this stands in contrast to our approach, where the CNN architecture is not at all the main focus and it is rather basic (i.e., 2 conv. layers followed each by a pool layer). Instead, our approach focuses on the design of the image representation, which does not require any specialized deep learning expertise for its use or further extension.

### 5.2. Comparison between Different Canvas Layouts

In order to account for the robustness of our approach, in this section we present the performance of our approach when modifying the baseline layout of the canvas that we used during all other experiments. This baseline layout is the one introduced in Section 3 and illustrated in Figure 4. The modifications are simple switching of halves or quadrants as specified in Table 10. The goal is to identify if the specific layout proposed as baseline is in fact relevant or if the regions can be moved around as long as the design is maintained consistent throughout the generation of the image representations. This experiment was conducted only for the WISDM dataset, since it is the simplest dataset in terms of the number of time series variables involved, which allows us to focus only on analyzing if the performance of the approach is greatly affected by the layout or not.

As we can see from the results in Table 10, the performance of our approach does not show much difference regardless of the specific layout used. Even for the case in which the quadrants are flattened over the rows of the canvas, and so the layout of the regions is modified almost completely, the performance of the approach remains very similar with respect to the case when the baseline layout is considered, with only a −0.0093 difference.

## 6. Discussion

### 6.1. Threats to Validity

The main threat to validity to our evaluation is that although we have used what we found to be the most widely recognized and used datasets in activity recognition from inertial sensors, the number of different activities considered is very constrained. This is because these datasets mostly consider very simple activities, such as walking and jogging, and overall they focus in the same activities or very similar ones, such as going up and down the stairs. Among the datasets, perhaps the one with activities that are different with respect to the ones in the other datasets is Skoda. However, on the downside it focuses on very specialized activities that might not correspond to activities performed by most users in real life scenarios. An inevitable workaround for this issue would be to collect a brand new dataset that includes a wider variety of scenarios and activities as well as a larger set of users.

### 6.2. Approach Limitations

An important limitation of our approach is that it requires some manual work related to the definition of the overall design used for the automatic generation of image representations. However, we have seen that, on the one hand, the manual work required for the configuration of such design is not heavier than the one required to configure a deep learning model, for which in addition specialized knowledge is needed. On the other hand, as we have observed in our experiments of Section 5.2, our approach is in general robust to changes in the configuration used in the design of the image representations. Therefore, we believe the manual work involved in our approach is considerably lower than its positives, particularly when compared to the work required in configuring complex ad hoc deep learning models.

### 6.3. Approach Practicality

In this section, we analyze the practicality of our approach, namely the challenges that one may encounter when taking it into a real-world application. In this respect, we believe that the biggest concern may be the impact on performance, specifically related to the efficiency with which a given activity can be recognized. In spite of this, we believe that our approach does not imply a big overhead. On the one hand, it is not hard to see that the algorithmic complexity of our image generation process is O(n), as the only operation involved is a sum of differences to compute the variation patterns over the time series. On the other hand, throughout our experiments, we found that the average time to generate an image representation is 0.044 seconds using a non-specialized GPU (we use Google Colaboratory’s basic GPU for all our computations), which we believe does not correspond to a major overhead when compared to the duration of the activities considered.

Another issue that may affect performance refers to the bottleneck caused by the use of nearby time windows to generate the image representations. We believe, however, that this could be alleviated by disregarding information from windows after the current window, in place of a model that improves continuously over time.

## 7. Conclusions

The recent success of deep learning in human activity recognition from inertial sensors has brought about many new approaches that have focused on improving the performance based mainly on proposing different architectures. In spite of this success, a relevant issue is related to how challenging may be to tune or developing further the increasingly involved deep learning models considered for activity recognition, which include ad hoc CNN and RNN architectures. In this paper, we have proposed an approach that automatically transforms inertial sensors time-series data into images that represent in pixel form patterns found over time, allowing even a simple CNN to outperform complex ad hoc deep learning models for activity recognition. We have evaluated our approach against the best performing approaches in the literature for 7 of the most widely known datasets in HAR. Our evaluation shows that our approach is able to achieve higher performance that the state of the art in all of these datasets and exhibits robustness across different configurations The main limitation of our approach is related to the work required to define the image representation designs. In spite of this, we have aimed at providing simple designs that are easy to implement and develop further.

## Figures and Tables

**Table 1:** Performance: our approach vs. other existent image representation approaches for Activity Recognition.

Approach	Dataset	Accuracy	Ours	Difference
Jiang and Yin [23]	UCI-HAR	0.9518	0.9930	+0.0412
	USC-HAD	0.9701	0.9900	+0.0199
Ignatov [24]	UCI-HAR	0.9760	0.9930	+0.0170
	WISDM	0.9332	0.9750	+0.0418
Jafariet al. [25]	PAMAP2	0.9800	0.9940	+0.0100

**Table 2:** Comparison considering WISDM dataset.

Approach	Details	Accuracy
Ignatov [24]	Time series treated as images + CNN	0.9332
Zeng et al. [37]	CNN-based approach	0.9475
Alsheikh et al. [19]	Deep learning models + HMMs	0.9446
Zhang et al. [38]	Based on U-Net network [39]	0.9640
Hossain et al. [30]	Deep and active learning models	0.9724
Our Approach	Image Representation of time series + CNN	**0.9750**

**Table 3:** Comparison considering UCI-HAR dataset.

Approach	Details	Accuracy
Qian et al. [40]	Distribution-Embedded Deep Neural Network (DDNN)	0.9058
Ronao and Cho [41]	CNN model	0.9460
Jiang and Yin [23]	DCNN using 2D activity image based on inertial signals	0.9518
Ignatov [24]	CNN + global statistical features	0.9760
Our Approach	Image Representation of Time series + CNN	**0.9930**

**Table 4:** Comparison considering USC-HAD dataset.

Approach	Details	Accuracy
Chen et al. [42]	Deep Long-Term Memory (LSTM)	0.9770
Jiang and Yin [23]	DCNN using 2D activity image	0.9701
Murad and Pyun [32]	LSTM-based deep RNN	0.9780
Our Approach	Image Representation of Time series + CNN	**0.9900**

**Table 5:** Comparison considering PAMAP2 dataset.

Approach	Details	*F*_1_-Score
Zeng et al. [36]	LSTM + Continuous Temporal	0.8990
Xi et al. [43]	Deep Dilated Convolutional networks	0.9320
Qian et al. [40]	Distribution-Embedded Deep NN (DDNN)	0.9338
Hammerla et al. [10]	CNN + RNN	0.9370
Jafari et al. [25]	CNN for multimodal time series image representations	0.9800
Our approach	Image Representation of Time series + CNN	**0.9900**

**Table 6:** Comparison considering Opportunity dataset.

Approach	Details	Value (Metric)
Hammerla et al. [10]	b-LSTM-S	0.9270 ($F_{1}$-score)
Xia et al. [44]	LSTM-CNN Architecture	0.9271 ($F_{1}$-score)
DeepConvLSTM [7]	Convolutional and LSTM network	0.9300 ($F_{1}$-score)
Hossain et al. [30]	Deep and active learning model	0.9406 (Accuracy)
Our approach	Image Representation of Time series + CNN	**0.9500** ($F_{1}$-score)
Our approach	Image Representation of Time series + CNN	**0.9540** (Accuracy)

**Table 7:** Comparison considering Daphnet dataset.

Approach	Details	Accuracy
Liu et al. [45]	Attention modules for spatial fusion	0.9094
Alsheikh et al. [19]	Deep learning models	0.9150
Qian et al. [40]	Distribution-Embedded Deep NN (DDNN)	0.9161
Hossain et al. [30]	Deep and active learning model	0.9234
Our approach	Image Representation of Time series + CNN	**0.9360**

**Table 8:** Comparison considering Skoda Mini checkpoint dataset.

Approach	Details	$F_{1}$-Score
Guan and Plötz [46]	Ensembles of deep LSTM networks	0.9260
AttnSense [47]	Attention with CNN and a GRU network	0.9310
Zeng et al. [36]	LSTM + Continuous Temporal Attention	0.9381
DeepConvLSTM [7]	Convolutional and LSTM network	0.9580
Our approach	Image Representation of Time series + CNN	**0.9970**

**Table 9:** Our approach vs. best approaches in the literature.

Dataset	Best Performing State-of-the-Art Approach				Our Approach	
	Paper	Approach	Metric	Result	Result	Difference
WISDM	Hossain et al. [30]	Deep and active learning	Accuracy	0.9724	0.9750	+ 0.0026
UCI-HAR	Zhang et al. [48]	Based on U-Net (28 conv. layers)	Accuracy	0.9840	0.9930	+ 0.0090
USC-HAD	Murad and Pyun [32]	DRNN Model	Accuracy	0.9780	0.9900	+ 0.0120
PAMAP2	Jafariet al. [25]	CNN (5 conv. layers) + image reps.	$F_{1}$-*score*	0.9800	0.9900	+ 0.0100
Opportunity	Ordóñez and Roggen [7]	Convolutional and LSTM network	$F_{1}$-*score*	0.9300	0.9530	+ 0.0230
Daphnet	Hossain et al. [30]	Deep and active learning	Accuracy	0.9234	0.9360	+ 0.0126
Skoda	Ordóñez and Roggen [7]	Convolutional and LSTM network	$F_{1}$-*score*	0.9580	0.9970	+ 0.0390

**Table 10:** Comparison between different canvas layouts.

Canvas Layout	Accuracy
baseline	0.9750
bottom & top halves switched	0.9572
flattened quadrants (over rows)	0.9657
left & right halves switched	0.9645
top-left & bottom-right quadrants switched	0.9519
top-right & bottom-left quadrants switched	0.9710
**Avg.**	**0.9642**
